# Diet and stable isotope analyses reveal the feeding ecology of the orangeback squid *Sthenoteuthis pteropus* (Steenstrup 1855) (Mollusca, Ommastrephidae) in the eastern tropical Atlantic

**DOI:** 10.1371/journal.pone.0189691

**Published:** 2017-12-15

**Authors:** Véronique Merten, Bernd Christiansen, Jamileh Javidpour, Uwe Piatkowski, Oscar Puebla, Rebeca Gasca, Henk-Jan T. Hoving

**Affiliations:** 1 GEOMAR Helmholtz Centre for Ocean Research Kiel, Kiel, Germany; 2 Universität Hamburg, Institute for Hydrobiology and Fishery Sciences, Hamburg, Germany; 3 Christian-Albrechts-Universität zu Kiel, Kiel, Germany; 4 El Colegio de la Frontera Sur, Chetumal, Mexico; Evergreen State College, UNITED STATES

## Abstract

In the eastern tropical Atlantic, the orangeback flying squid *Sthenoteuthis pteropus* (Steenstrup 1855) (Cephalopoda, Ommastrephidae) is a dominant species of the epipelagic nekton community. This carnivore squid has a short lifespan and is one of the fastest-growing squids. In this study, we characterise the role of *S*. *pteropus* in the pelagic food web of the eastern tropical Atlantic by investigating its diet and the dynamics of its feeding habits throughout its ontogeny and migration. During three expeditions in the eastern tropical Atlantic in 2015, 129 specimens were caught by hand jigging. Stomach content analyses (via visual identification and DNA barcoding) were combined with stable isotope data (∂^15^N and ∂^13^C) of muscle tissue to describe diet, feeding habits and trophic ecology of *S*. *pteropus*. Additionally, stable isotope analyses of incremental samples along the squid’s gladius—the chitinous spiniform structure supporting the muscles and organs—were carried out to explore possible diet shifts through ontogeny and migration. Our results show that *S*. *pteropus* preys mainly on myctophid fishes (e.g. *Myctophum asperum*, *Myctophum nitidulum*, *Vinciguerria* spp.), but also on other teleost species, cephalopods (e.g. Enoploteuthidae, Bolitinidae, Ommastrephidae), crustaceans and possibly on gelatinous zooplankton as well. The squid shows a highly opportunistic feeding behaviour that includes cannibalism. Our study indicates that the trophic position of *S*. *pteropus* may increase by approximately one trophic level from a mantle length of 15 cm to 47 cm. The reconstructed isotope-based feeding chronologies of the gladii revealed high intra- and inter-individual variability in the squid’s trophic position and foraging area. These findings are not revealed by diet or muscle tissue stable isotope analysis. This suggests a variable and complex life history involving individual variation and migration. The role of *S*. *pteropus* in transferring energy and nutrients from lower to higher trophic levels may be underestimated and important for understanding how a changing ocean impacts food webs in the eastern Atlantic.

## Introduction

Oceanic squids are muscular, fast moving opportunistic molluscan predators that feed on a variety of prey [1–3] to sustain high metabolic and growth rates [1,4–6]. Squids generally have a short lifespan of about 1–2 years and are semelparous; there is one reproductive cycle after which the individual dies [4]. They play a key role in the trophic structure of marine pelagic ecosystems [7,8] due to their large and in some areas increasing biomass [9] and their importance in the diet of marine predators such as fishes and marine mammals [10,11]. There is growing evidence that cephalopod populations may benefit from changing ocean environments and overexploitation of finfish species [9,12,13]. The capacity of oceanic squids (e.g. Humboldt squid) to take over niches of overexploited fish stocks and to flexibly adjust their distribution, life history and physiology may make them potential winners in a future ocean [14–16]. The impacts of squid population expansions on marine food webs are challenging to predict; thus, their ecological role needs to be better understood. In the eastern tropical Atlantic, the orangeback flying squid *Sthenoteuthis pteropus* (Ommastrephidae) is one of the dominant members of the epipelagic nekton community [17]. It is among the fastest growing squids and undertakes diel vertical migrations [18]. The species feeds in surface waters at night and migrates to deeper layers (down to 1200 m) during the day [19]. Information on their feeding ecology, including ontogenetic changes in diet and cannibalistic behaviours are limited [5,17–19]. The prey spectrum of *S*. *pteropus* switches during their ontogeny from crustaceans in early juveniles (3–9 cm mantle length (ML)) to micronektonic fishes in late juveniles and middle-sized individuals (9–35 cm ML) and finally to nektonic fishes and squids in adult large-sized squid (35–65 cm ML) [17,18]. This pattern is consistent with feeding in other ommastrephid squids [20–23]. Furthermore, *S*. *pteropus* is an important prey for top predators such as swordfish, marine mammals and sharks [17]. This influence of *S*. *pteropus* on high and low trophic levels in the ecosystem and its high abundance and reproduction rate suggests that they have a relevant role in the pelagic food web [17]. Stomach content analysis of hard parts that are resistant to digestion (e.g. squid beaks, fish otoliths and crustacean exoskeletons) allowed prey identification at different taxonomic levels [24]. Yet a limitation of the visual inspection of squid stomach contents is that prey items are often macerated beyond recognition or eroded due to digestion [1,25]. DNA sequencing of stomach contents can provide additional insights in prey species composition [26,27]. Since squids are known to reject hard parts, such as fish heads, stomach content analysis alone may bias when investigating prey species composition and prey size [1]. Additionally, the stomach contents represent only the last feeding event and do therefore not provide sufficient information about the squid’s average trophic position. The analysis of stable isotope (∂^15^N, ∂^13^C) ratios from body tissues has been used for studying the trophic role of squids and can provide time-integrated information about the trophic position of the recently assimilated (< 2 months) diet [28]. Tissue of consumers is enriched in ^15^N relative to their food, and therefore ∂^15^N values are indicators of a consumer’s trophic position [29]. In the marine environment, there is little variation in stable isotope ratios of carbon along the food chain, but carbon reflects spatial variations of the environment and can indicate inshore versus offshore, pelagic versus benthic feeding or latitudinal variations in foraging habitat [29]. Therefore, ∂^13^C may provide information about the animal’s foraging area, habitat and migration patterns. In order to obtain high resolution data on feeding ecology and individual migration behaviour, we analyzed stable isotope ratios of the cephalopod gladius. This archival hard structure, which consists of chitin and proteins, is present in the hatchlings and grows continuously with no metabolic turnover after synthesis. Using a combination of visual and molecular stomach content and stable isotope analysis we investigated the diet and the position of *S*. *pteropus* in the pelagic food web of the eastern tropical Atlantic and discussed size-based changes, individual variability of feeding habits, foraging habitats and migration.

## Materials and methods

**Fig 1:**
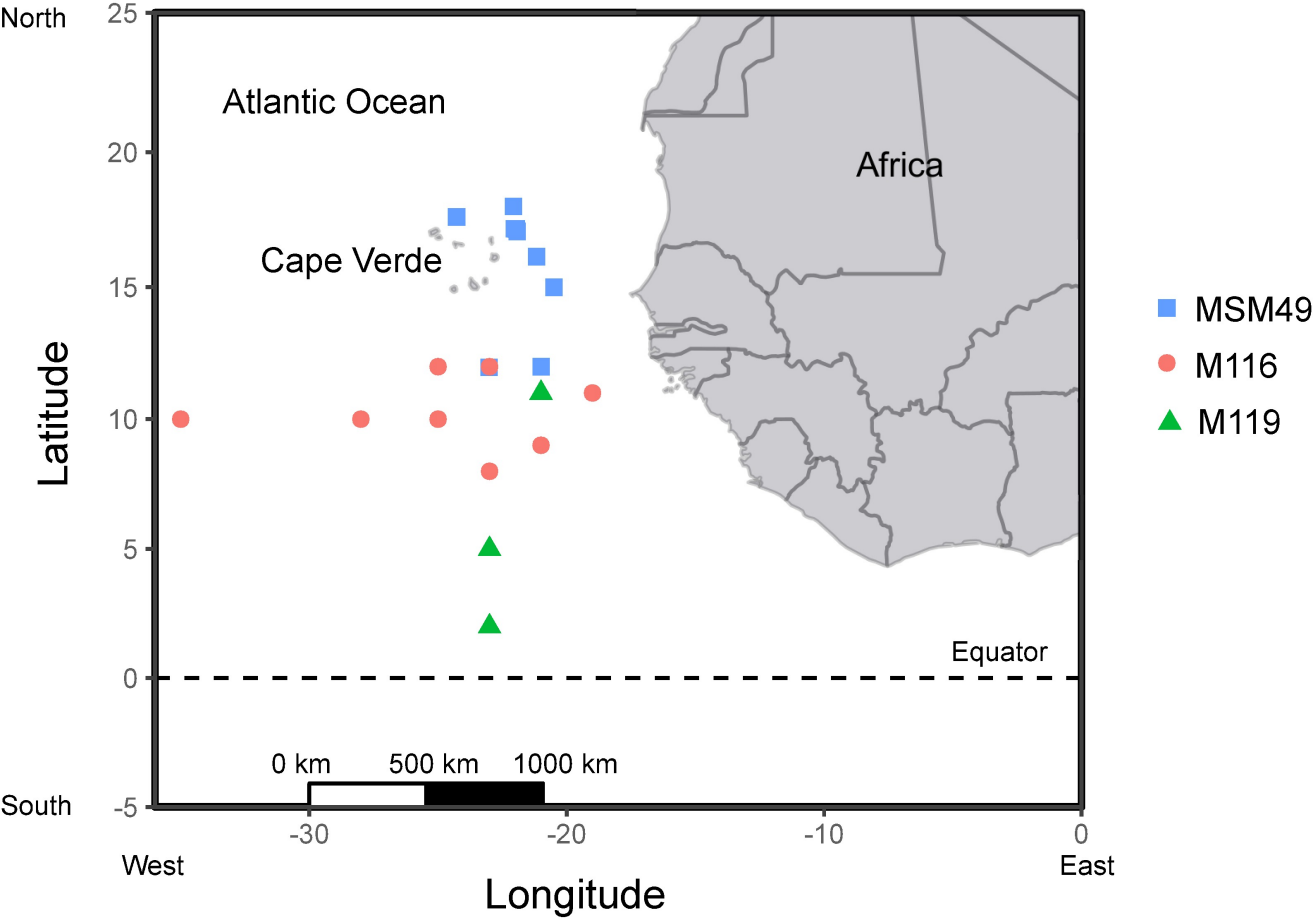
Capture locations of all *Sthenoteuthis pteropus* samples in the tropical eastern Atlantic in 2015 from three research cruises (MSM49, M116, M119).

Permission for sampling invertebrates in Cape Verdean waters was not required. A bilateral agreement between the Republic of Cape Verde and the Federal Republic of Germany grants German research vessels to conduct scientific research in Cape Verdean waters. The field studies did not involve sampling in protected areas or of endangered or protected species. Orangeback flying squid (n = 129) were caught in surface waters at night by hand jigging (jig size between 5–10 cm) with a light source for attraction. They were captured in the Cape Verde area of the eastern tropical Atlantic between 17°N– 2°N and 26°W– 21°W in May–June 2015 from the R/V Meteor (cruise M116), in September–October 2015 from the R/V Meteor (cruise M119) and in November–December 2015 from the R/V Maria S. Merian (cruise MSM49) (Fig 1). The squid were killed immediately after capture by decapitation [30]. Dorsal mantle length (ML) of all specimens were measured to the nearest millimeter and body mass was measured by a kitchen scale at calm sea or in the lab after defrosting. Sex and maturity stage were determined following Lipiński & Underhill [31]. A qualitative, visual stomach fullness index (SFI) was assigned: 0 = empty; 1 = traces of food; 2 = less than half full; 3 = more than half full; 4 = full; 5 = crammed with walls distended [32]. The stomachs of all individuals were stored in 70% ethanol or frozen (-40°C); samples of mantle muscle tissue and gladii from individuals of the cruise MSM49 were stored in -80°C and -40°C, respectively, for later stable isotope analysis.

### Stomach content analysis

Stomachs were defrosted, opened and contents screened through a 300 μm mesh sieve in order to sort prey items. A binocular stereomicroscope was used to identify prey items to the lowest possible taxon. Fish sagittal otoliths were identified following specialized regional literature [10,33–37]. Cephalopod beaks were identified according to Clarke [38], the Tree of Life Web Project [39] and a reference collection obtained during cruise WH383 around Cape Verde. Crustaceans were identified by their exoskeletons [40] and with the aid of the keys and illustrations posted at the Marine Species Identification Portal [41]. Frequency of occurrence and number were used to quantify occurrence of prey taxa in the stomachs [2,42]. The number of individual fishes or cephalopods that were found in one stomach were estimated by the maximum number of right or left otoliths and of upper or lower beaks. Frequency of occurrence (FO%) was calculated as the percentage of *S*. *pteropus* that fed on a certain prey and the relative number (N%) is the number of individuals of a certain prey, relative to the total number of individual prey.

### Stable isotope analysis

**Fig 2:**
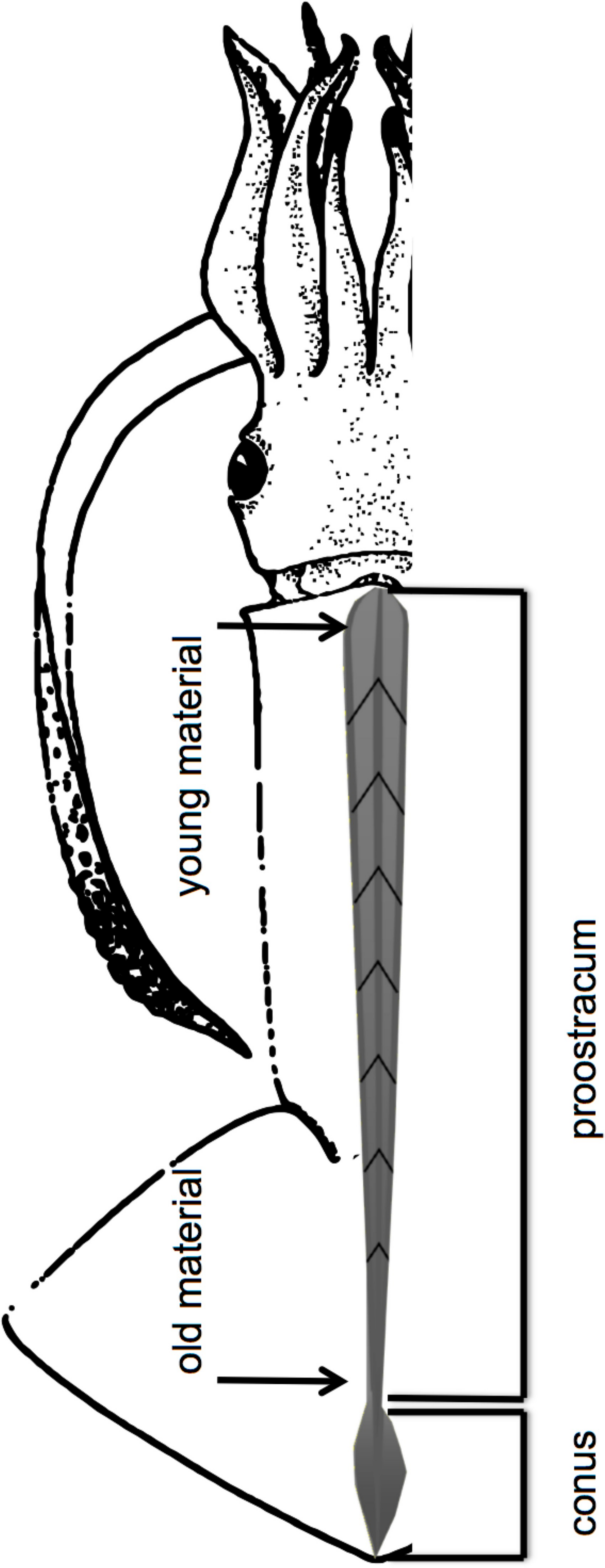
Gladius of a squid separated into a conus and proostracum (including rachis and vanes) section. (after Roper et al. [46] and Lorrain et al. [45]).

Stable isotope analysis was conducted only on the squid specimens caught in December 2015 (MSM49). Gladii were taken from the five largest individuals (five females > 40.0 cm ML, one male of 20 cm ML); muscle tissue was taken from 54 specimens (18.4–47.5 cm ML, females = 44; males = 10). Only the proostracum (including rachis and vanes) was used for stable isotope analysis (Fig 2), because its growth increments are clearly distinguishable and represent the entire lifespan of the squid [43–45]. Samples taken from the anterior part of the proostracum represent the most recently deposited organic material, while samples taken at the distal end correspond to the juvenile life phase.

Gladii were cleaned with distilled water, dried with KimWipes (Kimberly-Clark), measured to the nearest millimeter and the proostracum was cut in 10–20 mm increments following the V shape of the growth lines (Fig 2). The gladius samples (n_subsamples_ = 135) were freeze-dried for 24h and ground into a fine powder with mortar and pestle. The samples were freeze-dried again for 4h and weighed with a microbalance and 80–120 μg of the powder were transferred into tin containers for isotopic analysis. To estimate the current trophic position of the squid in the food web, stable isotopes (∂^13^C; ∂^15^N) were analyzed in muscle mantle tissue of 54 specimens including those that were used for gladii analysis. Muscle tissue samples were treated as the gladii. They were also freeze-dried for 24h and ground to a fine powder. However, lipids from muscle tissue samples were extracted using chloroform and the remaining tissue was dried over night at 50°C. Lipids were not extracted from the gladii samples because chitinous structures like beaks and gladii do not contain significant amounts of lipids that could bias ^13^C values [24]. The suggestion of Post et al. [47] was followed to conduct lipid corrections on ∂^13^C values if C/N ratios are higher than 3.5. This was the case for gladii ∂^13^C values. This method led to a small increase (1‰ in average) in ∂^13^C, but with a similar pattern over time (S1 Fig). Isotope ratios of C and N were measured using an elemental analyzer system (NA 1110, Thermo, Milan, Italy) connected to a temperature-controlled gas chromatography oven (SRI 9300, SRI Instruments, Torrance, CA, USA), which contained a column for permanent gases. Separated sample gases and the reference gases N_2_ and CO_2_ were transferred via a ConFloIII interface (Thermo Fisher Scientific, Bremen, Germany) to the isotope ratio mass spectrometer (Delta^Plus^ Advantage, Thermo Fisher Scientific). Measured isotope ratios are given as ∂ values in per mil deviation (‰) from the standard reference material Vienna PeeDee Belemnite (VPDB) and atmospheric nitrogen following the equation ∂X = [(R_Sample_/R_Standard_)-1]x1000 where X refers to ^13^C or ^15^N and R represents the ratio of the heavy isotope to the light isotope (^13^C/^12^C or ^15^N/^14^N). Laboratory gas cylinders of CO_2_ and N_2_ were used as working standards and calibrated against primary solid standards (IAEA-N1, -N2, -N3, USGS24, NBS22). The lab standard acetanilide used to estimate C and N content for each sample series was measured every seventh sample with a standard deviation of +/-0.16‰ for ∂^15^N and +/-0.39‰ for ∂^13^C.

### DNA barcoding

To complement visual stomach content analysis, a total of 27 unidentifiable tissue prey items from 23 *Sthenoteuthis pteropus* individuals (19.5–47.5 cm ML) and five crustaceans from the stomachs of 5 individuals were sequenced at mitochondrial cytochrome c oxidase subunit 1 (mCOI). The barcoded prey items represent a subset of all unidentifiable prey items of the stomachs. This approach provided expanded data of the squid’s prey spectrum and a way to detect cannibalism, because squids tend to tear their prey apart beyond recognition and spit out hard parts such as beaks which are difficult to swallow. Prey items were preserved in 70% ethanol after screening the stomach contents of *S*. *pteropus*. DNA was extracted with QIAGEN DNeasy Blood & Tissue columns following the manufacturer’s protocol. The COI barcode was amplified by a polymerase chain reaction (PCR) in 10 μL reactions containing 3μL DNA, 1 μL primer LCO1490 (F) and HCO21988 (R) for invertebrate samples [48] as well as VF2_t1 (F) and FR1d_t1 (R) for fishes [49] at 10μM concentration each, 0.1 μL Promega taq DNA Polymerase at 5U/μL concentration, 1 μL 10x PCR buffer, 1 μL dNTPs at 2mM concentration each and 4.9 μL sigma water. The PCR thermal cycle consisted of one initial denaturation step of 6 min at 96°C, 30 cycles of 20 s at 94°C, 30 s at 55°C and 40 s at 72°C, and one final extension step of 20 min at 72°C. PCR products were purified with ExoSAP-IT (Affymetrix USB) using 2 μl of cleanup reagent, incubated for 15 min at 37°C and inactivated for 15 min at 80°C. Sequencing reactions were performed in 10 μl volume containing 2 μl of purified PCR product, 1 μl primer at 10 μM concentration, 1 μl BigDye Terminator v3.1 (Applied Biosystems), 3 μl BigDye buffer and 3 μl sigma water. The PCR thermal cycle consisted of one initial denaturation step of 1 min at 96°C followed by 30 cycles of 10 s at 96°C, 5 s at 50°C and 4 min at 60°C. Sequencing reactions were run on an ABI PRISM 3130xl automated genetic analyzer (Applied Biosystems). Sequence trace files were exported into Codon Code Alligner (Codon Code Corporation) and the forward and reverse sequences were trimmed and assembled. Assembled contigs were examined and edited by hand. Consensus sequences were compared to public databases using the Basic Local Allignment Search Tool (BLAST) on the NCBI server (http://www.ncbi.nlm.nih.gov/blast)) and to reference cephalopod material obtained during the WH383 research cruise that took place in the same area.

### Data analysis

Data exploration to test for significant relationships and collinearity of explanatory variables of all regression analyses were conducted following the methods of Zuur et al. [50]. Regression analysis was used to determine whether stable isotope signatures of the muscle tissue changed with size. The largest individual (C) was treated as an outlier and excluded from the analysis, because it showed low ∂^15^N values throughout its entire life cycle, differing strikingly from the other individuals. A generalized additive model (GAM, R packages: nlme and mgcv [51,52]) was used to evaluate the relationship between ∂^15^N gladii stable isotope values as a function of gladii length. Since ∂^15^N gladii stable isotope values were not normally distributed, box-cox power transformation (lambda = 1.8) was applied to the data using the MASS package in R [53]. ∂^13^C data of gladii were corrected based on Post et al. [47]. Due to high level of residuals’ heterogeneity a generalized least squares (GLS) model with a fixed variance structure (VarFixed) was applied to test the ∂^13^C as a function of gladii length. To investigate the diet in relation to size, collected squid were divided into four size classes according to their mantle length. Squid ranging between 13.0 and 20.4 cm ML were defined as small, 20.5–30.4 cm as medium, 30.5–40.4 cm as large and 40.5–50.4 cm as very large. All statistics were performed with the freeware R (Version 3.3.2).

## Results

### Population structure

During the three research cruises a total of 129 specimens were captured with ML ranging between 13.1 and 47.5 cm; including 97 females (75%), 31 males (24%) and one unsexed individual (1%) (Table 1). Female mantle lengths ranged from 20.2 to 32.4 cm and body mass (BM) from 55 to 1,449 g and the male ML ranged from 17.5 to 21.7 cm and the BM from 151 to 327 g. Most females were immature (77%), followed by 13% mature and 6% maturing individuals. Male squid were mostly mature (75%), followed by 16% maturing and 9% immature individuals. The sample sizes were 22, 50 and 57 for cruises M119, M116 and MSM49, respectively.

**Table 1:** Population structure of *Sthenoteuthis pteropus* (n = 129) in the eastern tropical Atlantic in 2015 caught during three research cruises (latitude and longitude are rounded).

				Sex				
Cruise	Latitude (°N)	Longitude (°W)	Date	Female	Male	Juvenile	Total	ML (cm)
MSM49	17.4	24.1	December	6	0	0	6	21.5–30.5
	18.1	21.6	December	3	1	0	4	21.0–45.5
	17.1	22.0	December	4	6	0	10	18.4–34.6
	17.1	21.6	December	2	2	0	4	19.7–41.0
	17.1	21.5	December	3	0	0	3	21.5–47.5
	16.1	21.2	December	13	1	0	14	18.8–43.4
	14.6	20.3	December	3	0	0	3	26.3–36.8
	12.0	20.6	December	7	1	0	8	18.9–32.0
	11.6	23.0	December	4	1	0	5	18.7–30.8
M116	8.0	23.0	May/June	4	2	0	6	15.2–27.2
	9.0	21.0–40.0	May/June	2	0	0	2	21.1–27.9
	10.0	28.0	May/June	3	1	0	4	17.9–33.5
	10.0	25.0	May/June	5	4	0	9	15.6–39.0
	10.0	35.0	May/June	1	1	0	2	17.1–25.3
	11.0	19.0	May/June	5	0	0	5	20.4–25.7
	12.0	23.0	May/June	5	4	1	10	15.5–33.1
	12.0	22.0	May/June	5	1	0	6	18.0–27.1
	12.0	25.0	May/June	5	1	0	6	17.9–30.5
M119	11.0	21.0	Sept./Oct.	3	1	0	4	19.5–25.0
	2.0	23.0	Sept./Oct.	14	4	0	18	13.1–31.0
			**Total**	**97**	**31**	**1**	**129**	**13.1–47.5**

### General diet analysis

Few stomachs were crammed with food (3%). Most stomachs were either full (29%), half full (27%) or less than half full (23%). Traces of food were observed in 10% of all squid stomachs and 8% were empty (S2 Fig). Stomach contents showed three main groups of prey: fishes, cephalopods and crustaceans (Table 2). A total of 336 fish otoliths and 52 cephalopod beaks were found. Over 80% of cephalopod and crustacean occurrences were single and no more than three individuals per stomach occurred. 50% of fish occurrences were single or double and more than five individuals per stomach were rare. A stomach contained on average 3 prey species ± 1.9 with 9 prey species being the maximum found in one stomach.

**Table 2:** Summary of prey composition found in the stomach contents of *Sthenoteuthis pteropus* from the eastern tropical Atlantic in 2015 by frequency of occurrence (FO), frequency of occurrence in percent (%FO), number (N) and number in percent (%N).

Prey	FO	%FO	N	%N
Pisces	88	67.7	336	82.8
Myctophidae	76	58.5	208	51.2
*Bolinichthys* sp.	5	3.8	6	1.5
*Ceratoscopelus warmingii*	10	7.7	12	3.0
*Diogenichthys atlanticus*	1	0.8	1	0.2
*Diaphus* sp.	1	0.8	1	0.2
*Diaphus dumerilii*	5	3.8	12	3.0
*Diaphus fragilis*	2	1.5	2	0.5
*Diaphus lucidus*	1	0.8	1	0.2
*Diaphus vanhoeffeni*	9	6.9	9	2.2
*Gonichthys* sp.	4	3.1	4	1.0
*Hygophum* sp.	7	5.4	9	2.2
*Hygophum hygomii*	12	9.2	13	3.2
*Hygophum macrochir*	7	5.4	9	2.2
*Hygophum taaningi*	11	8.5	21	5.2
*Hygophum proximum*	3	2.3	4	1.0
*Hygophum reinhardtii*	3	2.3	9	2.2
*Lampanyctus* sp.	3	2.3	4	1.0
*Lampanyctus intricarius*	1	0.8	1	0.2
*Lampanyctus festivus*	1	0.8	1	0.2
*Lampanyctus nobilis*	2	1.5	2	0.5
*Lepidophanes gaussi*?	1	0.8	1	0.2
*Lepidophanes guentheri*	1	0.8	1	0.2
*Myctophum* sp.	10	7.7	16	3.9
*Myctophum asperum*	18	13.8	26	6.4
*Myctophum nitidulum*	15	11.5	21	5.2
*Myctophum obtusirostre*	1	0.8	1	0.2
*Myctophum selenops*	4	3.1	4	1.0
*Myctophum spinosum*	4	3.1	4	1.0
*Notoscopelus caudispinosus*	1	0.8	3	0.7
*Symbolophorus* sp.	4	3.1	5	1.2
*Symbolophorus rufinus*	4	3.1	5	1.2
Unidentified Myctophidae	18	13.8	22	5.4
Other Pisces				
Bathylagidae	3	2.3	4	1.0
Bregmacerotidae	4	3.1	5	1.2
*Bregmacerotidae* sp.	1	0.8	1	0.2
*Merluccius* sp.	3	2.3	4	1.0
Exocoetidae	4	3.1	6	1.5
*Exocoetus* sp.	3	2.3	3	0.7
*Exocoetus obtusirostris*	2	1.5	3	0.7
Nomeidae	1	0.8	2	0.5
*Cubiceps pauciradiatus*	1	0.8	2	0.5
Paralepididae	2	1.5	4	1.0
*Paralepididae* sp.	1	0.8	1	0.2
*Lestidiops sphyrenoides*	2	1.5	2	0.5
*Lestrolepis intermedia*	1	0.8	1	0.2
Phosichthyidae	9	6.9	41	10.1
*Vinciguerria attenuata*	4	3.1	21	5.2
*Vinciguerria nimbaria*	8	6.2	20	4.9
Stomiidae	1	0.8	2	0.5
*Chauliodus sloani*	1	0.8	2	0.5
Unidentified Otoliths	41	31.5	64	15.8
**Cephalopoda**	**37**	**28.5**	**52**	**12.8**
Bolitaenidae	2	1.5	2	0.5
Enoploteuthidae	8	6.2	9	2.2
Histioteuthidae	1	0.8	1	0.2
Mastigoteuthidae	1	0.8	1	0.2
Octopoda (cirrata)	2	1.5	2	0.5
Ommastrephidae	2	1.5	2	0.5
Onychoteuthidae	1	0.8	1	0.2
Pyroteuthidae	1	0.8	1	0.2
Unidentified Beak (destroyed or upper beak)	25	19.2	33	8.1
**Crustacea**	**17**	**13.1**	**18**	**4.4**
Decapoda	14	10.8	15	3.7
Euphausiacea	3	2.3	3	0.7
**Total**	**142**		**406**	

**Fig 3:**
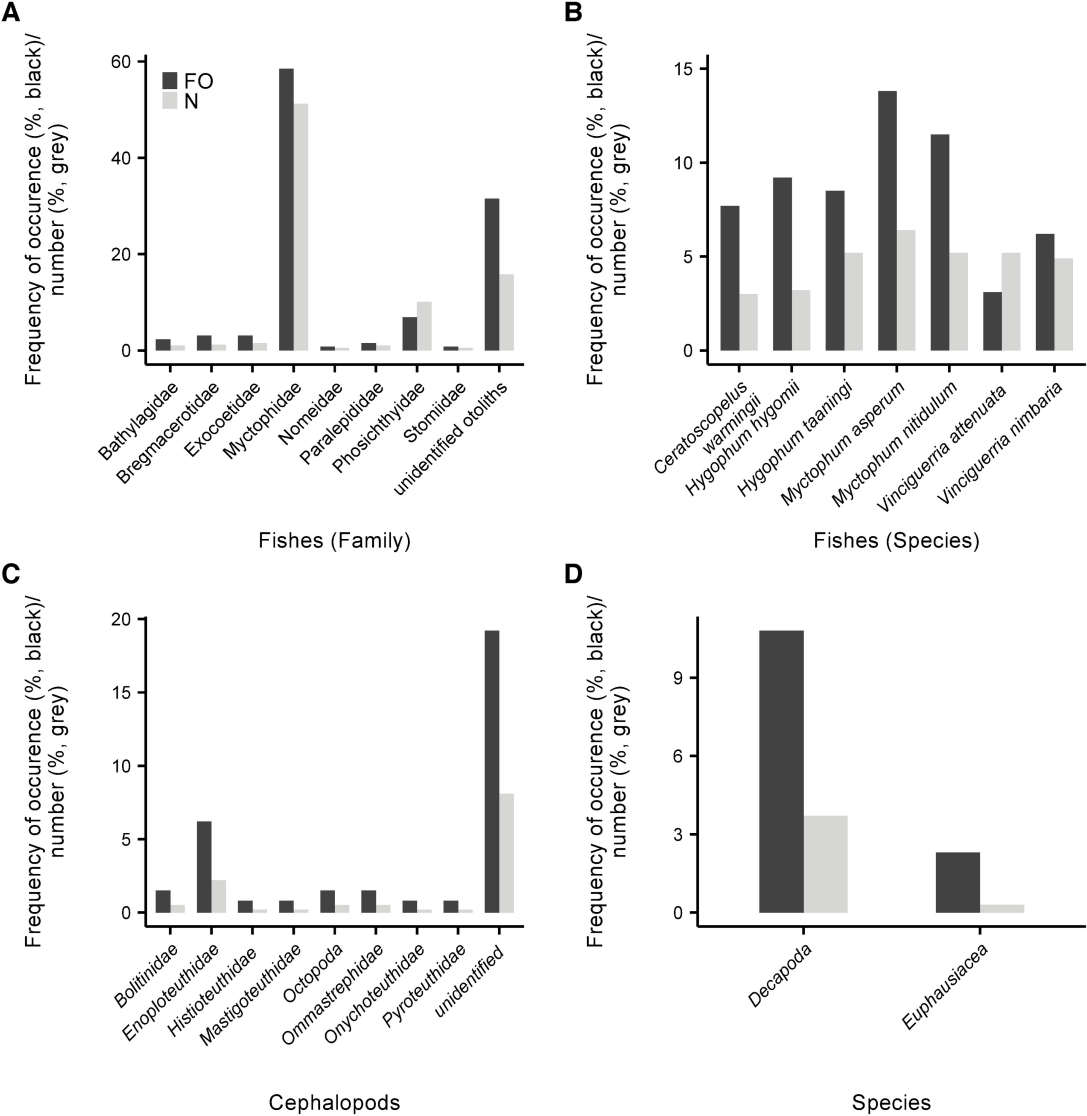
Frequency of occurrence (FO, black bars) and relative number (N, grey bars) in percent of the stomach contents of *Sthenoteuthis pteropus* (n = 129) caught in the eastern tropical Atlantic in 2015. (A) Fish families (B) Fish species (only a subset of the most abundant species is shown) (C) Cephalopod families (D) Crustaceans.

**Fig 4:**
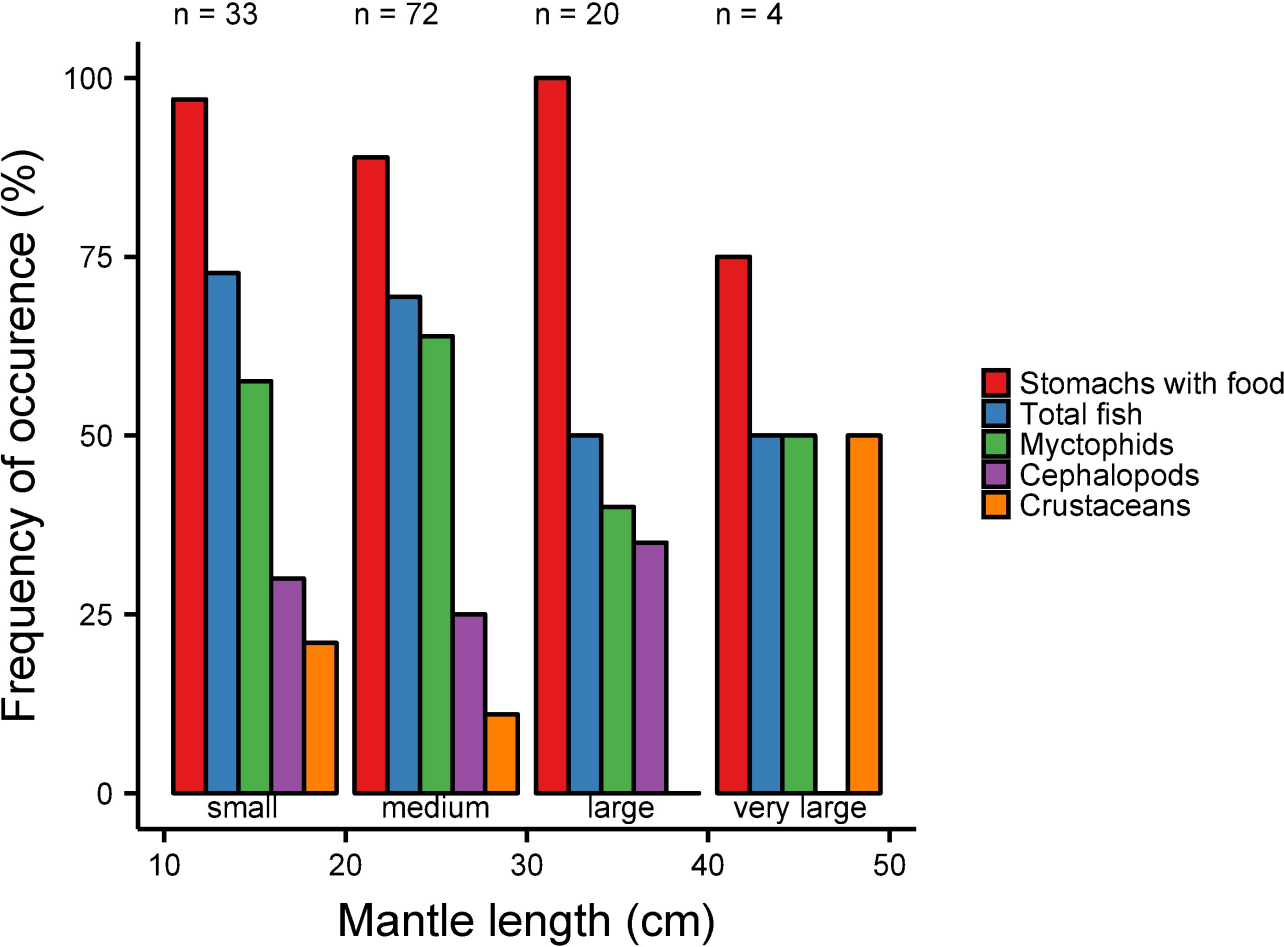
Frequency of occurrence of the prey groups of 129 specimens of *Sthenoteuthis pteropus* for 10 cm size intervals. Sample size per size interval: small (11–20 cm) = 33; middle (21–30 cm) = 72; large (31–40 cm) = 20; very large (41–50 cm) = 4.

About 68% of the stomachs examined contained fish, accounting for 83% by prey number. The most abundant prey family in terms of both occurrence and numbers were Myctophidae (58.5% FO and 51.2% N) (Fig 3A) and this was independent of the squid size (Fig 4). Seven of the eight largest individuals (> 35 cm, included in the size classes large and very large) had only myctophids in their stomachs and one stomach contained flying fish (Exocoetidae). Because the sample size for the largest size group is low (n = 4), the diet results of very large sized squid have to be interpreted with caution. While 30 different myctophid species were identified in total, *Myctophum asperum* and *Myctophum nitidulum* most frequently occurred in the stomachs (13.8% FO and 11.5% FO, respectively) (Fig 3B). Fish otoliths that could not be identified accounted for 15.8% N, thus representing an occurrence of 31.5% FO. Fishes of the genus *Vinciguerria* (Phosichthyidae) occurred in 6.9% of all stomachs and accounted for 10.1% of all fish prey. They were usually not abundant in the diet of small and middle-sized squid, occurring in only 6–15% of stomachs and not consumed by large-sized squid (Fig 4). However, in 9 stomachs, they were present in high numbers (n = 41). Other fish families found as squid prey were Bathylagidae, Bregmacerotidae, Paralepididae, Stomiidae and Nomeidae (Fig 3A). The maximum number of individual fishes counted in one stomach was 22 and these were the species *V*. *attenuata* and *V*. *nimbaria* (in a female squid, ML 23.5 cm).

Cephalopods were the second most important prey group of *S*. *pteropus*, occurring in 29% of all stomachs and accounting for 13% of all prey by number (Table 2). Since the beaks were mainly very small and eroded by digestion, they could only be identified to family level. In contrast to fish otoliths, squid beaks consumed by *S*. *pteropus* were not dominated by a single taxonomic group. Cephalopod prey were moderately abundant (24–35%) in the stomachs of *S*. *pteropus* smaller than 40 cm (n = 125) and absent in very large-sized squid (n = 4) (Fig 4).

Approximately 2% of the lower beaks were identified as Enoploteuthidae and were present in 6% of all stomachs. Cirrate octopods made up 0.5% of all crustacean prey and occurred in 1.5% of all stomachs. Besides that, cephalopods from the Bolitinidae, Ommastrephidae, Pyroteuthidae, Mastigoteuthidae, Histioteuthidae and Onychoteuthidae family were present in the stomachs with abundances and occurrences below 2% for each family (Fig 3C). Beaks that could not be identified accounted for the largest grouping and were present in 19% of all stomachs and made up 8% of all prey. 13% of all stomachs contained crustaceans, mainly decapods (11%) and euphausiids (2%) (Fig 3D). Decapods could not be identified to species level due to the advanced stage of digestion. Crustaceans had the lowest frequency of occurrence (11–21%) in small- to middle-sized squid and 50% in very large-sized squid

(Fig 4). Crustaceans were not found in stomachs of large-sized squid (n = 20). Of all examined stomachs, 20 included copepods with a total of 198 copepods overall. A total of ten amphipod individuals were found in eight stomachs (Table 3). There was no evidence of recently ingested large prey that could have introduced copepods and amphipods as secondary or transitory prey in the stomachs of *S*. *pteropus*. The copepods and amphipods occurred almost exclusively in squid caught during May and June (M116). The mantle length of squid containing more than one intact copepod specimen ranged from 15 to 25 cm. The maximum number of copepods found in one individual was 83 (female, ML = 25 cm). All amphipods belong to the suborder Hyperiidea (n = 10). Three specimens were identified as *Vibilia* spp. (Vibiliidae), one as *Hyperietta vosseleri* (Lestrigonidae), two as members of the Phronimoidea and two as Platysceloidea.

**Table 3:** Summary of secondary prey found in the stomach contents of *Sthenoteuthis pteropus* from the eastern tropical Atlantic in 2015 by frequency of occurrence (FO) and number (N). Secondary prey refers to prey that has been introduced in the stomachs by other prey species.

Secondary Prey	FO	N
Crustacea	31	212
**Amphipoda (all suborder Hyperiidae)**	10	10
Vibiliidae		
*Vibilia* spp.	3	3
Lestrigonidae		
*Hyperietta vosseleri*	1	1
Phronimoidea	2	2
Platysceloidea	2	2
unidentified hyperiid amphipod	2	2
**Cladocera**	3	4
**Copepoda**	20	198
**Other**	**4**	**10**
**Chaetognatha**	1	3
**Pteropoda**	2	6
**Algae**	1	1
**Total**	**35**	**222**

### DNA barcoding

The BLAST analyses provided generally low E values, high query covers and high percent identities (Table 4). Ingested cephalopod prey included *Sthenoteuthis pteropus* (Ommastrephidae; n = 5), *Enoploteuthis leptura* (Enoploteuthidae; n = 1) and *Histioteuthis reversa* (Histioteuthidae; n = 2); fish prey included *Lestidium atlanticum* (Paralepididae; n = 1), *Cheilopogon* sp. (Exocoetidae; n = 2), Hemiramphidae (n = 1) and *Myctophum affine/nitidulum* (Myctophidae; n = 1). Crustacean prey included the hyperiid amphipods *Vibilia* sp. (Vibiliidae) and *Hyperietta vosseleri* (Lestrigonidae). Additionally, copepods of the genus *Temora* sp. (Temoridae) and *Labidocera* sp. (Pontellidae) were identified. Samples that could not be assigned with high confidence to a species were excluded from the analysis. Cannibalistic specimens ranged between 19.0 and 30.5 cm ML.

**Table 4:** Sequenced samples of prey items collected in the stomachs of *Sthenoteuthis pteropus* in the tropical eastern Atlantic in 2015.

Order		Family	Highest hit identified by BLAST	Query cover	Identity	E value	Total score	Sequence length
Squids								
Oegopsida		Enoploteuthidae	*Enoploteuthis leptura*	97%	99%	0.0	1027	589
Teuthida		Ommastrephidae	*Sthenoteuthis pteropus*	100%	97%	4,00e-173	618	366
Teuthida		Ommastrephidae	*Sthenoteuthis pteropus*	100%	99%	0.0	1067	594
Teuthida		Ommastrephidae	*Sthenoteuthis pteropus*	100%	99%	0.0	806	440
Teuthida		Ommastrephidae	*Sthenoteuthis pteropus*	100%	99%	0.0	922	531
Teuthida		Ommastrephidae	*Sthenoteuthis pteropus*	100%	100%	9.00 e-47	200	108
Teuthida		Histioteuthidae	*Histioteuthis reversa*	96%	95%	0.0	502	596
Teuthida		Histioteuthidae	*Histioteuthis reversa*	100%	94%	0.0	852	548
Fishes								
Aulopiformes		Paralepididae	*Lestidium atlanticum*	99%	98%	0.0	1040	622
Beloniformes		Exocoetidae	*Cheilopogon sp*.	100%	98%	0.0	672	391
Beloniformes		Exocoetidae	*Cheilopogon sp*.	100%	98%	0.0	966	556
Beloniformes		Hemiramphidae	Hemiramphidae *sp*.	100%	93%	0.0	865	579
Myctophiformes		Myctophidae	*Myctophum affine/nitidulum*	100%/99%	99%/ 97%	0.0	973/919	552
Crustaceans								
Amphipoda		Vibiliidae	*Vibilia spec*.	100%	85%	6,00e-179	608	549
Amphipoda		Lestrigonidae	*Hyperietta vosseleri*	90%	88%	0.0	719	628
Calanoida		Temoridae	*Temora stylifera*	100%	99%	5.00 e-86	327	186
Calanoida		Pontellidae	*Labidocera sp*.	99%	82%	3.00 e-131	479	479
Calanoida		Temoridae	*Temora stylifera/* *discaudata*	94%/ 93%	89%/ 85%	0.0	1059/655	625
Reference collection								
Oegopsida		Enoploteuthidae	*Enoploteuthis leptura*	96%	85%	0.0	645	600
Teuthida		Ommastrephidae	*Sthenoteuthis pteropus*	98%	97%	0.0	1040	645
Teuthida		Histioteuthidae	*Histioteuthis reversa*	100%	94%	0.0	948	594

### Stable isotope analysis

#### Stable isotope analysis of muscle tissue

**Fig 5:**
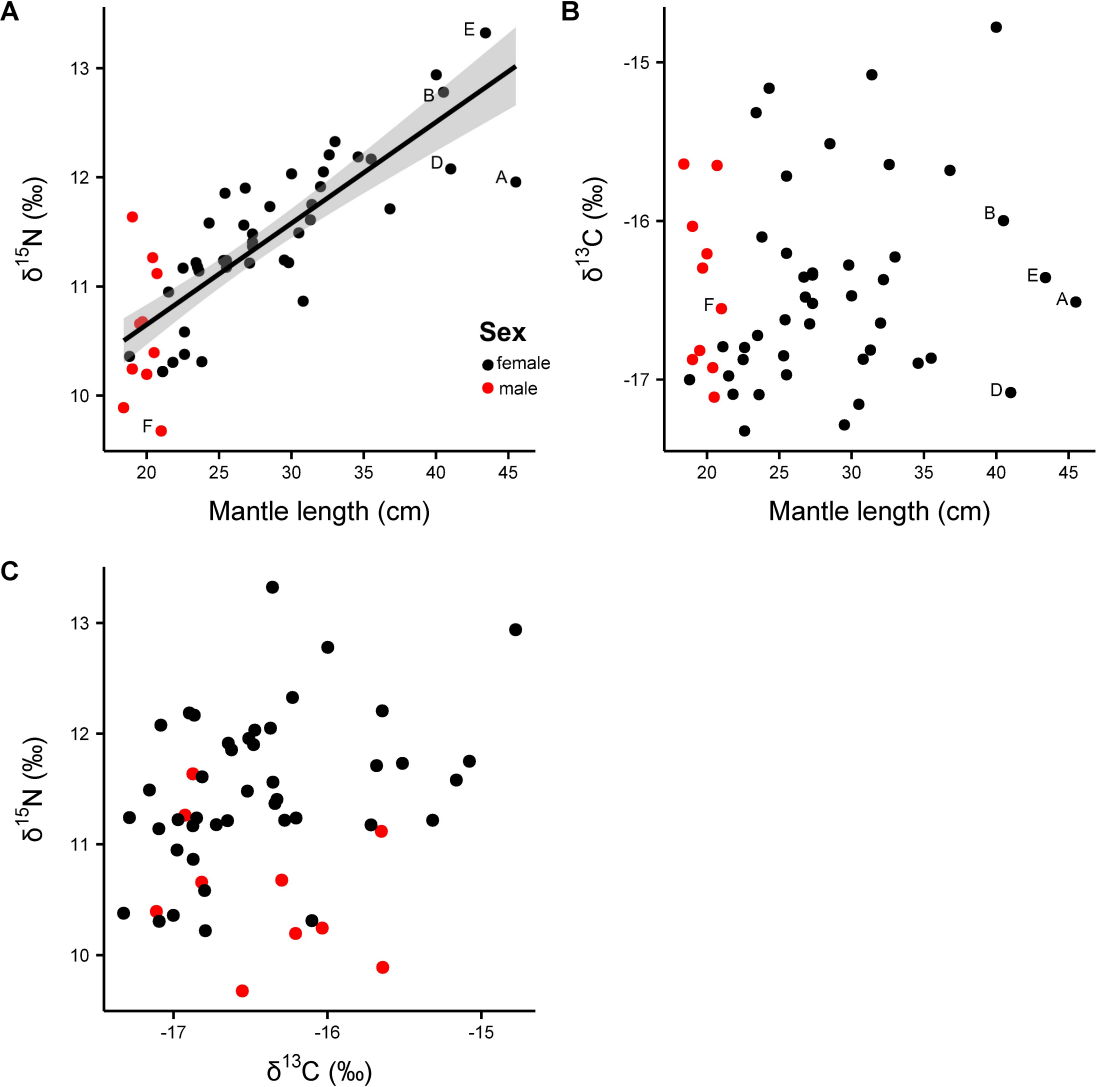
Isotopic values of muscle tissue (n = 54) in relation to mantle length of *Sthenoteuthis pteropus* caught in the eastern tropical Atlantic in 2015. (A) ∂^15^N in ‰ of muscle tissue; the smoother curve (method = GAM) was adapted by the function y ~ s (x, k = 4); the shaded area is the 95% confidence interval for predictions; (B) ∂^13^C ‰ of muscle tissue; without regression line since this one was not significant; (C) Gender specific biplot of stable isotope values of muscle tissue (n = 54) of *Sthenoteuthis pteropus* caught in the eastern tropical Atlantic in 2015; The labeled data points (A–F) correspond to the individuals on which gladii stable isotope analysis was applied (see Table 5).

Muscle ∂^13^C isotope values ranged from -17.3 to -14.8‰ (difference: 2.5‰) and ∂^15^N ranged from 9.7 to 13.3‰ (3.6‰) (Fig 5, Table 5). The difference of 3.6‰ in ∂^15^N in muscle tissue equals an increase by one trophic level [54]. A significant effect of size (ML) was found for muscle ∂^15^N isotope values (y = 8.796 + 0.093x, r = 0.67, F_1, 51_ = 102.1, p < 0.001) showing an increase by 2.5 ‰ in ∂^15^N with increasing ML (Fig 5A). No significant relationships were found between ∂^13^C muscle isotope values and sex, maturity stage and location (∂^13^C: p = 0.50, r^2^ = 0.18, F_10,43_ = 0.95) and no relationship between ∂^13^C and mantle length could be observed (p = 0.39, r^2^ = 0.01, F_1, 52_ = 0.74). For ∂^15^N and sex, maturity stage and location a marginally significant relationship was found (∂^15^N: p = 0.07, r^2^ = 0.31, F_10,43_ = 1.92) attributed to the fact that male individuals of *S*. *pteropus* do not grow as large as females and therefore occupy lower size classes with lower ∂^15^N values (Fig 5A).

**Table 5:** Size, location of capture, isotope values and C/N mass ratios of the five large *Sthenoteuthis pteropus* females and the small male individual caught during cruise MSM49 in 2015 in the eastern tropical Atlantic.

				**Mantle**			**Gladius**								
				**∂**^**15**^**N**	**∂**^**13**^**C**	**C/N**	**∂**^**15**^**N**			**∂**^**13**^**C**			**C/N**		
**Individual**	**Size [cm]**	**Latitude (°N)**	**Longitude (°W)**				**mean**	**max**	**min**	**mean**	**max**	**min**	**mean**	**max**	**min**
A	45.5	18.0835	22.0007	11.9	-16.5	3.7	7.0	8.0	6.2	-16.9	-15.7	-17.8	4.5	4.7	4.3
B	40.5	17.1622	21.9218	12.8	-16.0	3.4	7.6	8.0	7.3	-16.4	-16.1	-17.3	4.5	4.7	4.4
C	47.5	17.1622	21.9218	11.3	-16.9	3.7	6.5	8.6	5.3	-16.7	-16.0	-17.1	4.5	4.6	4.4
D	41.0	17.1853	21.9612	12.1	-17.1	3.8	7.8	8.3	7.4	-16.6	-16.0	-16.9	4.4	4.8	4.3
E	43.4	16.2032	21.3085	13.3	-16.4	3.6	8.3	9.2	7.5	-16.1	-15.4	-17.5	4.4	4.6	4.0
F	21.0	18.0835	22.0007	9.7	-16.6	3.5	6.5	7.6	5.9	-16.7	16.4	-17.2	4.6	4.6	4.5

#### Stable isotope analysis of squid gladii

**Fig 6:**
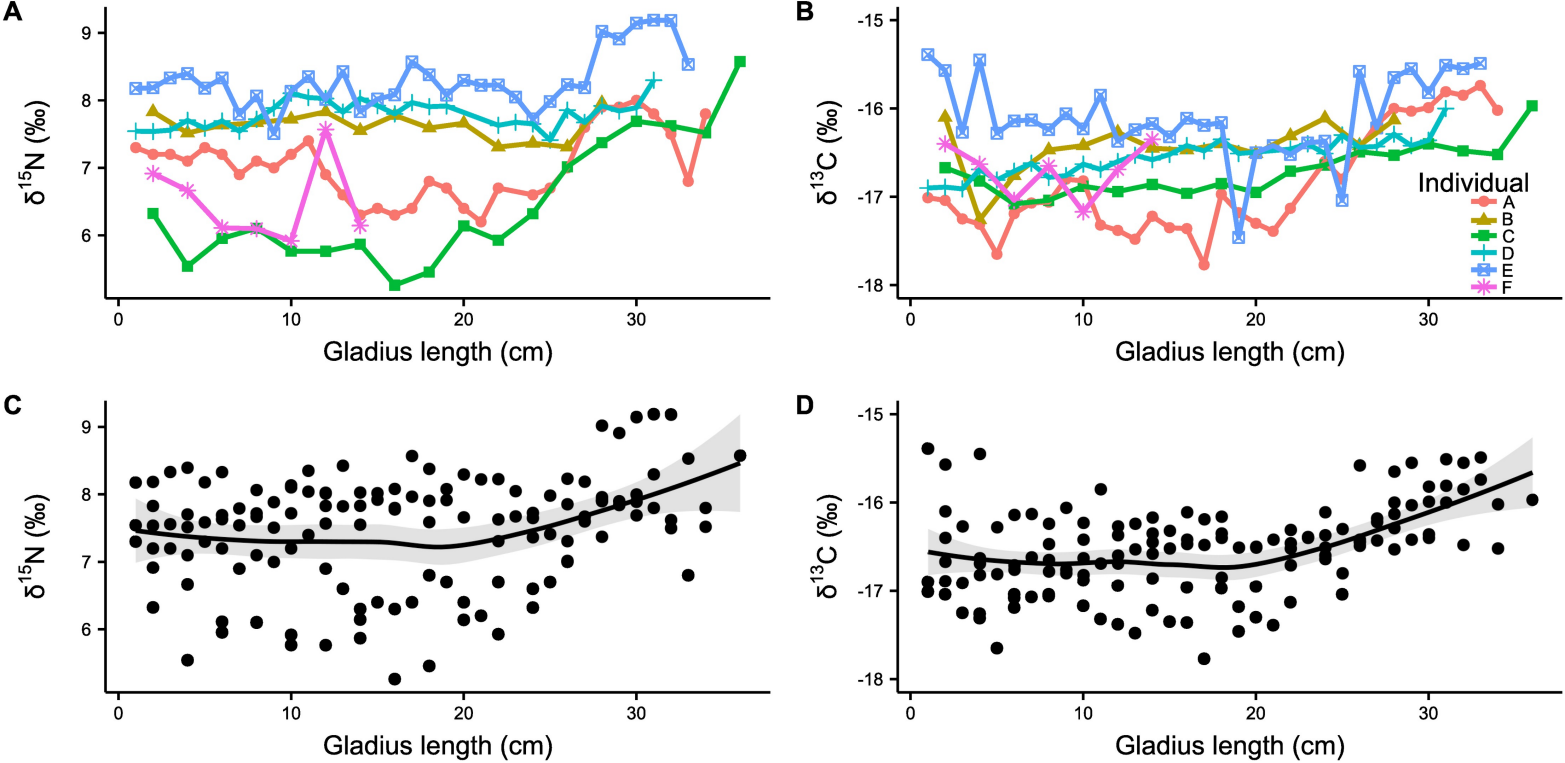
Stable isotope values along the gladius length. (A) ∂^15^N and (B) ∂^13^C stable isotope values of the five large female *Sthenoteuthis pteropus* (A–E) and the small male individual (F) caught in the eastern tropical Atlantic in 2015. (C) Grouped ∂^15^N values (D) Grouped ∂^13^C values. Lines represent significant relationships (∂^15^N: p < 0.05; ∂^13^C: p < 0.01).

Ranges of ∂^15^N and ∂^13^C gladii stable isotopes were 5.3–9.2‰ (range: 3.9‰) and -17.8–15.4‰ (range: 2.4‰), respectively (Table 5). Single specimens showed different regimes of ∂^15^N and ∂^13^C (Fig 6). However, grouping of the gladii stable isotope data showed a significant increase in ∂^15^N and ∂^13^C with increasing gladius length (Fig 6C and 6D, GAM_∂_^15^_N_, edf = 2.4, F = 2.8, p < 0.05, r^2^ = 0.14; GLS_∂_^13^_C_, t = 3.9, p < 0.01). The largest squid analyzed (Individual C) showed a total increase in ∂^15^N of 3.3‰ from the earliest gladii section to the most recent (5.3‰ ∂^15^N at 16 cm GL to 8.6‰ ∂^15^N at 36 cm GL), accompanied by an increase of 1‰ in ∂^13^C (-17.0‰ ∂^13^C at 16 cm GL to -16.0‰ ∂^13^C at 36 cm GL). Individual A showed fluctuating isotopic values with decreasing and increasing ∂^15^N and ∂^13^C. Opposite to individual A, individual D did not show high fluctuations in ∂^15^N (range between 7.4 and 8.3 ‰); instead a steady increase of ∂^13^C from -16.9 ‰ to -16‰ was observed. The ∂^15^N and ∂^13^C values of all individuals revealed that the trophic position and foraging habitat varied at short time intervals over their entire life span. However, all large individuals showed the same pattern of ∂^15^N and ∂^13^C which slowly increased after reaching a GL of approximately 20–25 cm. The isotopic values of the male individual (individual F) were in the range of females and also showed high variability. No relationship was found between stable isotope values in gladii and muscle tissue and the location of capture.

## Discussion

The present study on the feeding ecology of *Sthenoteuthis pteropus* revealed three major findings. 1) Stomach content data obtained during 2015 showed that juvenile and adult *S*. *pteropus* mainly prey on myctophids, but that they also show an opportunistic and variable feeding behavior. No ontogenetic size related diet shift in prey composition was detected; this was probably because of the small sample size of large squid. 2) The muscle tissue stable isotope analysis showed an overall increase in ∂^15^N corresponding with the growth of the squid (assuming a constant isotopic baseline). The ∂^13^C isotopic values did not show any trend with increasing ML and therefore indicated no consistent change in migration behaviour with growth, thus suggesting that individuals have different foraging areas.

3) The reconstruction of the feeding chronology of individual squid via incremental gladii stable isotope analysis did not reveal a continuous increase in trophic position during the squid’s entire life. However, a significant increase in ∂^15^N and ∂^13^C was observed when squid exceeded ca. 20 cm GL which corresponded to the isotope data from the muscle tissue. Furthermore, gladius analyses suggested substantial individual variations in trophic positions and foraging area.

### Stomach content analysis–visual and DNA analysis

The diet of *Sthenoteuthis pteropus* consisted mainly of myctophid fishes, which is also the main prey item of many other ocean squids including gonatids [55] and ommastrephids [56,57]. A total of 30 different myctophid species were found in the stomachs of *S*. *pteropus*, but *Myctophum asperum* and *M*. *nitidulum* dominated. These species are among the most abundant myctophid fishes that undertake diel vertical migration in the tropical and sub-tropical Pacific [58] but little information is available on these species in the Atlantic [59]. *Myctophum asperum* and *M*. *nitidulum* can reach a maximal length of 8.5 cm and 8.3 cm, respectively and prey on small crustaceans such as copepods and amphipods [58,60]. Adults of both species feed mainly in the epipelagic zone at night within the upper 1 m layer and descend to deeper layers during the day; thus they represent a relevant role in the transfer of energy from sea surface layers to the deep. Late juveniles and adult squid rise to the epipelagic layer at night to forage (0–150 m) and descend down to 800–1200 m in the morning [17]. By doing so *S*. *pteropus* actively transports carbon from the upper ocean layers into deeper regions. Individual squid had up to nine species of myctophids in their stomachs, stressing the diverse prey spectrum of *S*. *pteropus*. Such diversity of prey species is known from other squid species [3] and could be explained by the migratory behavior of *S*. *pteropus*. However, many different myctophid species are able to coexist due to resource-partitioning of vertical distribution and diet [61,62]. Therefore the diversity in prey species could also be due to co-existence of different myctophid species in the same habitat. The high number of prey taxa of *S*. *pteropus* indicates that this squid is an opportunistic predator. Besides fishes, *S*. *pteropus* also preyed upon cephalopods and crustaceans. A dietary shift during growth from crustacean-dominated prey to fish and cephalopod-dominated prey was not apparent from the stomach contents. This was probably due to the small sample size of large squid (4 very large and 125 small to large individuals) and the squid’s opportunistic feeding behavior. The dominance of fishes and squids in the stomachs examined might be related to their local abundance and availability as potential prey items, but also that *S*. *pteropus* selectively predate on these groups. When cephalopods and crustaceans were found in squid stomachs they mostly occurred as single individuals, whereas fish remains were found in higher numbers. This suggests that either *S*. *pteropus* feeds on fish schools or that fish otoliths accumulate in the stomachs over several meals. The latter would result in a biased frequency of occurrence and number [56]. DNA barcoding revealed that *S*. *pteropus* feeds on conspecifics. This finding was not observed in the visual investigation of the stomach contents because beaks were too small and eroded to be identified to species level. Contamination is unlikely since *S*. *pteropus* sequences were not systematically found in all samples, and were the only sequence that did amplify when found. Furthermore, only very clean sequences were analyzed and the dissection kit was cleaned after each sample. Cannibalism has been reported for several cephalopods [4,55]. Cannibalistic behaviour can provide a competitive advantage among juveniles and/or adults during episodes of food scarcity [63] and can be a regulating factor to reduce intra-specific competition [64]. Cannibalism could also be artificially induced by jig fishing [55] as has been shown in Humboldt squid which occurs in high abundances in the Pacific Ocean [56]. During our fishing operations we only observed squid in very small schools and therefore the observed cannibalism is likely a natural component of the feeding behaviour of *S*. *pteropus* as was suggested for gonatid squid [55].

### Zooplankton as prey

High numbers of copepods and some hyperiid amphipods were found in some of the examined squid stomachs. The crustaceans were intact, undigested and there were no fish or crustaceans present in the stomachs that could have introduced them to the squid’s stomachs as secondary or transitory prey. It is unlikely that the squid actively predates individually on these copepods and hyperiids. Some of the encountered amphipods and copepods are known to be symbionts or prey of gelatinous zooplankton [65–67]. Therefore, these crustaceans could have entered the stomachs of *S*. *pteropus* with the gelatinous fauna, suggesting that squid had been feeding on gelatinous zooplankton (e.g. salps, medusae, siphonophores, pyrosomes). Ingested gelatinous zooplankton is subject to rapid digestion, a process that continues after capture even when specimens are being frozen [68]. In the eastern tropical Atlantic *S*. *pteropus* has been found to feed on pyrosomes where they were abundant [69]. Almost all amphipods found in the stomachs of *S*. *pteropus* belong to the suborder Hyperiidae. Members of the hyperiid genus *Vibilia* sp. are well-known symbionts of salps [65,66]. One amphipod belonging to the superfamily Phronimoidea also associates with salps, ctenophores, scyphozoans and antho- and leptomedusan hydrozoans [66,70–73]. One other encountered amphipod was assigned to Platysceloidea and this superfamily mostly associates with siphonophores or in some cases with medusae [70,71,74]. High abundances of gelatinous zooplankton [75] as well as cephalopods [76] have been found in the equatorial upwelling region and subtropical and tropical waters of the Atlantic, respectively, where our samples were taken. Gelatinous zooplankton play an important role in energy and matter transformation and its direct importance as prey may be largely underestimated [69,77]. Even though it is of low caloric value due to its high water content, a large predator may satisfy parts of its energy requirements by preying on large amounts of gelatinous zooplankton [78] and focusing on body parts of higher energetic value such as gonads or stomachs. Furthermore, the low energy content may be compensated for by faster digestion [68]. Although gelatinous zooplankton taxa are increasingly recognized as an important prey for higher trophic predators [78–80], only few accounts exist to date for cephalopods [68,81–84]. Our findings present a good case for why it is probable that *S*. *pteropus* is also consuming gelatinous zooplankton (i.e. salps, medusa) in the eastern tropical Atlantic.

### Stable isotope analysis of squid gladii

The stable isotope analysis of *S*. *pteropus* gladii provided a broad picture of its feeding ecology. Our results showed not only an increase from lower to higher trophic level prey in some individuals, but also strong individual variation in all squid throughout their entire life. Although all individuals showed different ∂^15^N baselines as juvenile squid, ∂^15^N in gladii tissue increased significantly with a GL > 20 cm. The individuals C and E showed a particularly steep increase. These findings are in line with the mantle stable isotope measurements reported in this study, showing an increase in ∂^15^N by 2.5 ‰ from 15.0 to 47.5 cm ML. The high variation in nitrogen stable isotopes observed in all individuals could be explained by movements into foraging areas with different isotopic baseline values since this species is highly migratory [17]. *Sthenoteuthis pteropus* is able to temporarily live in a pronounced oxygen minimum zone (OMZ) [5,17,19] and undergoes intense vertical migration [17]. In the absence of oxygen_,_ bacteria use nitrate to consume organic matter (denitrification). Denitrification preferentially removes ^14^N-NO_3_^-^ and leaves residual nitrate ^15^N-enriched [85] which leads to an increase in the baseline ∂^15^N [29,86]. Additionally, ∂^15^N values of marine predators are affected by vertical migration. Predators feeding on mesopelagic prey resources have higher ∂^15^N values than predators feeding on epipelagic prey [87,88] possibly as an effect of nutrient cycling [89–91]. Therefore, variation in ∂^15^N can only be interpreted as a shift in trophic position when the squid does not change foraging area (no change in ∂^13^C), because such a change may affect the ∂^15^N baseline [29]. Without a ∂^15^N baseline we cannot clearly distinguish between an increase in trophic position and an increase in ∂^15^N baseline values due to horizontal or vertical migration. The ∂^13^C values of the most recent gladius increments of the five large squid (A, B, C, D and E) were similar suggesting that they foraged in the same habitat before capture. However, during their lifetime ∂^13^C values fluctuated substantially in all individuals and increased significantly after 20 cm GL. *Sthenoteuthis pteropus* spawns in the eastern equatorial Atlantic and its early life stages are quickly dispersed in the equatorial zone [17,19]. Females from the northern population (north of equator) migrate about 2500 km during the summer from the Cape Verde Islands up to Madeira and back [19]. Immature and mature females form several large groups in different geographical ranges with immature females occupying colder waters and mature females inhabiting warmer waters [19]. Zuev and Nikolsky [19] identified two distinct size groups of mature females in the same region where we sampled: an equatorial and a northeastern group. From December until May these two groups merge and in June until November they separate again [19]. These differences in migratory behavior may explain the differences in ∂^13^C and ∂^15^N in the gladii of the five individuals throughout almost their entire life and suggest that they foraged in different habitats with different isotopic baselines. The ∂^13^C signature follows productivity. It shows higher values in productive nearshore waters, such as upwelling zones. In less productive offshore regions, the ∂^13^C signature has lower values. In pelagic ecosystems the ∂^13^C signatures are lower at higher latitudes than at lower latitudes. [29]. All females analyzed in this study were caught at low latitudes close to a productive upwelling zone. This could explain the significant increase in ∂^13^C in individuals larger than 20 cm GL. However, many of the prey of *S*. *pteropus* also migrates and hence are also affected by different stable isotope baselines, whose signatures then manifests in the predatory squid. Analyses of squid and prey isotope signatures from multiple years and seasons are needed to draw a full picture of *S*. *pteropus* trophic ecology, identify environmental effects and trace down the causes of variation in stable isotope signatures.

### Individual variation in *Sthenoteuthis pteropus*

Although several studies stress that individual ecology varies widely among species and populations [45,88,92,93], traditionally, conspecific individuals are considered to be ecologically equivalent. In this study, gladii isotopic values showed strong intra- and inter-individual variation over time and body size. Overlapping ∂^13^C and ∂^15^N values were observed among some individuals, indicating foraging in similar habitats and at similar trophic levels, but every examined squid revealed a unique isotopic pattern throughout its life. The variable and strong intra-individual variation in isotopic shifts that were observed along the proostracum are in accordance with other studies (*Dosidicus gigas* and *Berryteuthis magister)* suggesting a complex life history of these squids [3,28,45,94]. Squid may conform their foraging strategies to prey availability that changes with season, year and habitat. Spatial and temporal variation in prey availability combined with phenotypic differences between *S*. *pteropus* individuals may shift the squid populations from generalists to foraging specialists [3,95,96]. This hypothesis is supported by the findings of Ruiz-Cooley et al. [21] and Hunsicker et al. [3], which suggest that the variable but increasing ∂^15^N values along the proostracum of *Dosidicus gigas* and *Berryteuthis magister* are an effect of prey availability and optimal foraging strategy. The increasing but variable ∂^15^N values suggest that *S*. *pteropus* opportunistically feeds on available prey [17], but as it grows it becomes able to consume prey from higher trophic levels. For example, ∂^15^N in muscle from *Ommastrephes bartramii* over 4 years differed significantly, possibly due to changes in prey consumption [97]. The largest female (Individual C; 47.5 cm ML) investigated in this study had 1 to 2‰ lower ∂^15^N isotopic values in muscle tissue than the other four large females (> 40 cm ML). Its trophic position (with ∂^15^N of 11.3 ‰) seems to be similar to squid smaller than 30 cm. Stable isotope data from muscle would have lead us to incorrectly assume that squid individual C occupies the lowest trophic position compared with the other four large individuals. However, gladii data showed that ∂^15^N values of all squid were different throughout their life and individual C hatched in a region with the lowest ∂^15^N baseline of all the large individuals investigated. This finding emphasizes individual variation that can only be detected by applying multiple techniques. Individual variation as observed here may have been underestimated in previous studies on feeding in oceanic squid, but may have potentially important ecological, evolutionary and conservation implications [92,93].

### Stable isotope analysis of muscle

∂^15^N values of the muscle tissue of *S*. *pteropus* had a range of 3.6‰, with a significant increase by around 2.5 ‰ as the squid grows to a ML of around 40 cm. This is the equivalent to an increase of one trophic level [54]. Since this species can reach a ML of about 65 cm [17], it is likely that its ∂^15^N values would continue to increase when growing larger than 40 cm. These findings are in accordance with previous studies on oceanic squids e.g. *Dosidicus gigas*, *Ommastrephes bartramii*, *Todarodes filippovae* and *Berryteuthis magister* which show an increase of one trophic level in ∂^15^N by ~4 ‰, ~ >5‰, ~3‰ and 3.5 ‰, during ontogeny respectively [3,21,22,97]. The findings of our study have to be interpreted with caution, since this shift was not detected in the stomach content analysis, probably because of the small sample size of large squid. Additionally, the observed increase in ∂^15^N could also be due to squid migrating into areas with different isotopic baselines or an increase in foraging depth, facilitated by the growing swimming capacities of adult squid. ∂^13^C values did not show any trend with increasing body size, revealing individual differences in foraging areas with no consistent migration pattern; a trend that was also seen in other studies [3,22]. In the open ocean a considerable part of predation pressure on fish stocks may originate from epipelagic ommastrephid squids. Their role as predators and their transfer of energy and nutrients from the mesopelagic food web to higher trophic levels may be underestimated (1). Furthermore, squids cope well with changing ocean environments (9) that are detrimental for other species. The eastern tropical Atlantic is characterized by a pronounced oxygen minimum zone (OMZ) (65,66) which is expanding due to global warming and eutrophication (67,68). *Sthenoteuthis pteropus* is adapted to temporarily live in environments with low oxygen concentrations by anaerobic metabolism (5) and active migration (17), whereas OMZ expansion reduces the habitat for fast swimming fishes (69). The continuing depletion of predatory fish communities (70) may reduce predation pressure on juvenile *S*. *pteropus* in the eastern tropical Atlantic. How the eastern tropical Atlantic population of *S*. *pteropus* responds to this ongoing environmental and ecological changes needs to be subject for further research.

## Supporting information

S1 Fig Corrected and uncorrected ∂^13^C values of the gladii tissue stable isotope analysis of *Sthenoteuthis pteropus* caught in 2015 Lipid corrections on ∂13C values of gladii tissue according to Post et al. 2007. (PDF)

S2 Fig Overview of the stomach fullness indices of *Sthenoteuthis pteropus* from 2015 caught during the cruises MSM49, M119 and M116. (EPS)
