# Supplementary figures and images for: Diet and stable isotope analyses reveal the feeding ecology of the orangeback squid Sthenoteuthis pteropus (Steenstrup 1855) (Mollusca, Ommastrephidae) in the eastern tropical Atlantic
Source: PLoS One. 2017 Dec 15;12(12):e0189691. doi: 10.1371/journal.pone.0189691 (PMC5731754; doi:10.1371/journal.pone.0189691)

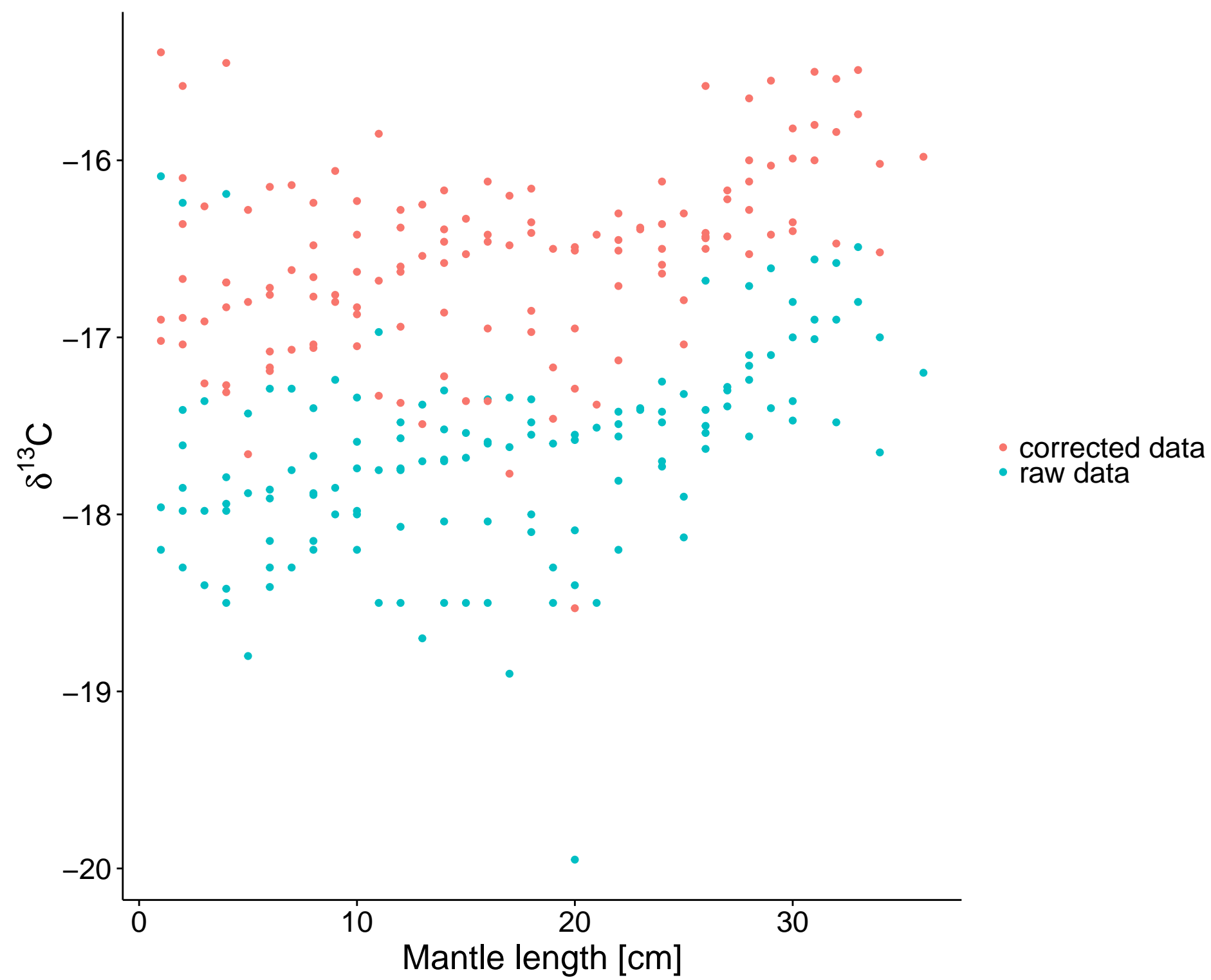

Supplement: S1 Fig — (PDF) [file pone.0189691.s001.pdf]
